# SEraster: a rasterization preprocessing framework for scalable spatial omics data analysis

**DOI:** 10.1093/bioinformatics/btae412

**Published:** 2024-06-20

**Authors:** Gohta Aihara, Kalen Clifton, Mayling Chen, Zhuoyan Li, Lyla Atta, Brendan F Miller, Rahul Satija, John W Hickey, Jean Fan

**Affiliations:** Center for Computational Biology, Whiting School of Engineering, Johns Hopkins University, Baltimore, MD 21211, United States; Department of Biomedical Engineering, Johns Hopkins University, Baltimore, MD 21218, United States; Center for Computational Biology, Whiting School of Engineering, Johns Hopkins University, Baltimore, MD 21211, United States; Department of Biomedical Engineering, Johns Hopkins University, Baltimore, MD 21218, United States; Center for Computational Biology, Whiting School of Engineering, Johns Hopkins University, Baltimore, MD 21211, United States; Department of Biomedical Engineering, Johns Hopkins University, Baltimore, MD 21218, United States; New York Genome Center, New York, NY 10013, United States; Center for Computational Biology, Whiting School of Engineering, Johns Hopkins University, Baltimore, MD 21211, United States; Department of Biomedical Engineering, Johns Hopkins University, Baltimore, MD 21218, United States; Center for Computational Biology, Whiting School of Engineering, Johns Hopkins University, Baltimore, MD 21211, United States; Department of Biomedical Engineering, Johns Hopkins University, Baltimore, MD 21218, United States; New York Genome Center, New York, NY 10013, United States; Center for Genomics and Systems Biology, New York University, New York, NY 10003, United States; Department of Biomedical Engineering, Duke University, Durham, NC 27708, United States; Center for Computational Biology, Whiting School of Engineering, Johns Hopkins University, Baltimore, MD 21211, United States; Department of Biomedical Engineering, Johns Hopkins University, Baltimore, MD 21218, United States

## Abstract

**Motivation:**

Spatial omics data demand computational analysis but many analysis tools have computational resource requirements that increase with the number of cells analyzed. This presents scalability challenges as researchers use spatial omics technologies to profile millions of cells.

**Results:**

To enhance the scalability of spatial omics data analysis, we developed a rasterization preprocessing framework called SEraster that aggregates cellular information into spatial pixels. We apply SEraster to both real and simulated spatial omics data prior to spatial variable gene expression analysis to demonstrate that such preprocessing can reduce computational resource requirements while maintaining high performance, including as compared to other down-sampling approaches. We further integrate SEraster with existing analysis tools to characterize cell-type spatial co-enrichment across length scales. Finally, we apply SEraster to enable analysis of a mouse pup spatial omics dataset with over a million cells to identify tissue-level and cell-type-specific spatially variable genes as well as spatially co-enriched cell types that recapitulate expected organ structures.

**Availability and implementation:**

SEraster is implemented as an R package on GitHub (https://github.com/JEFworks-Lab/SEraster) with additional tutorials at https://JEF.works/SEraster.

## 1 Introduction

Spatial omics technologies enable high-throughput molecular profiling of single cells or small groups of cells while preserving their spatial relationships within tissue sections (Bressan *et al.* 2023). This high-throughput profiling demands computational analysis to leverage both molecular and spatial information in extracting relevant biological insights. Various computational tools have been developed for such analysis, ranging from those that identify spatially variable genes (SVGs) (Svensson *et al.* 2018, Sun *et al.* 2020, Kats *et al.* 2021, Miller *et al.* 2021, Zhu *et al.* 2021, Weber *et al.* 2023) to those that delineate spatial organization and interactions between different cell types (Shao *et al.* 2022, Cang *et al.* 2023, Kim *et al.* 2023, Li *et al.* 2023, Peixoto *et al.* 2023). However, many of these computational tools have runtime and memory requirements that increase with the number of single cells or spatial points analyzed, presenting challenges as technologies continue to improve and researcher apply them to generate large-scale spatial omics data with millions of spatial points. Therefore, preprocessing to streamline such large-scale spatial omics data analyses is needed.

A similar scalability challenge previously emerged for the analysis of single-cell RNA sequencing (scRNA-seq) datasets. To address this problem, preprocessing frameworks were developed to subsample cells while maintaining representative transcriptional heterogeneity (Hie *et al.* 2019, Ren *et al.* 2019) or aggregate transcriptionally similar cells into metacells prior to downstream analysis (Baran *et al.* 2019, Bilous *et al.* 2022). For spatially resolved data specifically, self-organizing maps have also been applied to aggregate neighboring single-cells into nodes that preserve the topological relations and relative densities of the sample (Hao *et al.* 2021). These preprocessing techniques, by either subsampling or aggregating, reduce the number of cells analyzed, thereby lessening the computational resource requirements of downstream analysis and enhancing scalability.

Here, to enhance the scalability of spatial omics data analysis, we developed a preprocessing framework called SEraster to aggregate spatially proximal cells into pixels using rasterization prior to downstream analysis. SEraster further implements sparse matrix representations and parallel processing for enhanced efficiency. We benchmarked the performance of SEraster on downstream spatial omics data analyses including identifying SVGs and cell-type co-enrichment to demonstrate that SEraster enables scalable and accurate analysis of large-scale spatial omics datasets through integration with existing spatial omics data analysis tools. SEraster is implemented as an R package (available on GitHub https://github.com/JEFworks-Lab/SEraster) and with SeuratWrappers (available on GitHub https://github.com/satijalab/seurat-wrappers), which are amenable to SpatialExperiment and Seurat infrastructures, respectively, for storing spatial omics data, allowing for streamlined integration with existing spatial omics analysis tools.

## 2 Materials and methods

**Figure 1.**
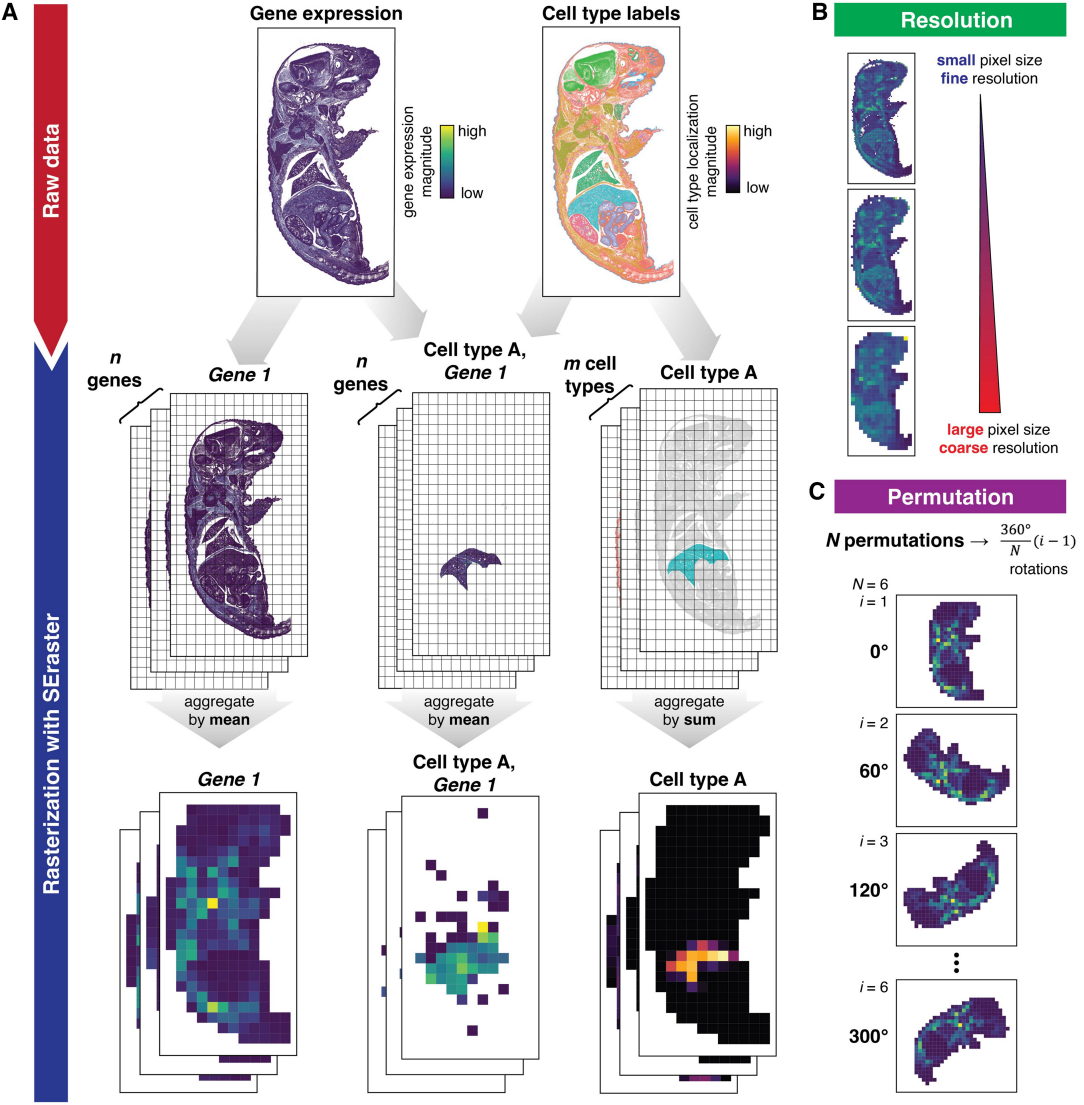
Overview of SEraster. (A) SEraster reduces the number of spatial points in a given spatial omics dataset prior to downstream analysis by rasterizing or aggregating single cells’ gene expression (using mean) or cell-type labels (using sum) into equally sized pixels. SEraster can be applied to aggregate gene expression in a label-specific (e.g. cell-type or cluster) manner as well. (B) SEraster allows users to control the rasterization resolution or the side length of the pixel. Finer resolution corresponds to a smaller pixel size and coarser resolution corresponds to a larger pixel size. Plots are colored by total gene expression per pixel (aggregated using mean). (C) SEraster enables permutation by rotating the dataset at various angles before rasterization. Downstream analyses performed on rotated datasets can be summarized to help control for edge effects. Plots show total gene expression per pixel (aggregated using mean)

SEraster reduces the number of spatial points in spatial omics datasets for downstream analysis through a process of rasterization where single cells’ gene expression or cell-type labels are aggregated into equally sized square or hexagonal pixels based on a user-defined resolution (Fig. 1A, Supplementary Information S1). Here, we refer to a particular resolution of rasterization by the side length for square pixels and the distance between opposite edges for hexagonal pixels such that finer resolution indicates smaller pixel size and vice versa (Fig. 1B). To create a rasterized representation, SEraster initially employs the sf package (Pebesma *et al.* 2018) to generate pixels by defining square or hexagonal grids that span the *x* and *y* spatial coordinate values in the spatial omics dataset. Square pixels are used by default and for all subsequent analyses. For continuous variables such as gene expression or other molecular information, SEraster aggregates the observed raw counts or normalized expression values for each molecule within each pixel using means by default. Such rasterization can also be performed in a cell-type-specific manner by restricting to cells of a particular cell-type prior to rasterization. Alternatively, to create a rasterized representation of categorical variables such as cell-type or cluster labels, SEraster first converts the labels to a model matrix using a one-hot encoding and then treats the model matrix as a features-by-observations matrix to aggregate the number of cells for each label within each pixel using sums by default. In general, the aggregation function can be chosen to accommodate user-defined purposes. This rasterization process is implemented in a pixel-wise manner, which is optionally parallelized with the BiocParallel package (Morgan *et al.* 2023). Compared to other R packages that perform rasterization on vector data represented as dense matrices such as terra (Hijmans *et al.* 2024) or stars (Pebesma *et al.* 2023), SEraster rasterizes spatial omics datasets represented as either dense or sparse matrices with the Matrix package (Bates *et al.* 2024). Since the features-by-observations matrix and model matrix are often sparse, this feature further allows SEraster to reduce resource requirements upon rasterization preprocessing. In addition, since rasterized values may be sensitive to edge effects such as the specific boundaries of grids upon rasterization, SEraster enables permutation by rotating the dataset at various angles before rasterization (Fig. 1C, Supplementary Information S2). The rasterized output is returned as a SpatialExperiment object, allowing for streamlined integration with existing spatial omics analysis tools within the R/Bioconductor framework (Righelli *et al.* 2022) for downstream analyses. SEraster is also implemented as a SeuratWrappers (SeuratWrappers Contributors 2024) to run directly on Seurat objects, enabling further analysis following existing Seurat spatial pipelines.

To explore the potential utility of rasterization in spatial omics analysis, we apply SEraster as a preprocessing step prior to downstream spatial omics analysis using both simulated and real spatial omics data. In particular, we characterize the impact of rasterization on runtime and benchmark accuracy in identifying SVGs with the nnSVG package (Weber *et al.* 2023) as compared to other down-sampling approaches. We further demonstrate how rasterized cell types can be used with the CooccurrenceAffinity package (Mainali and Slud 2022, Mainali *et al.* 2022) to recapitulate expected pairs of cell types that tend to be spatially co-enriched. In this manner, SEraster can be used as a preprocessing step to enable scalable and accurate analysis of large-scale spatial omics datasets with existing tools.

## 3 Results

### 3.1 Rasterization reduces runtime while maintaining accuracy in the identification of spatially variable genes

**Figure 2.**
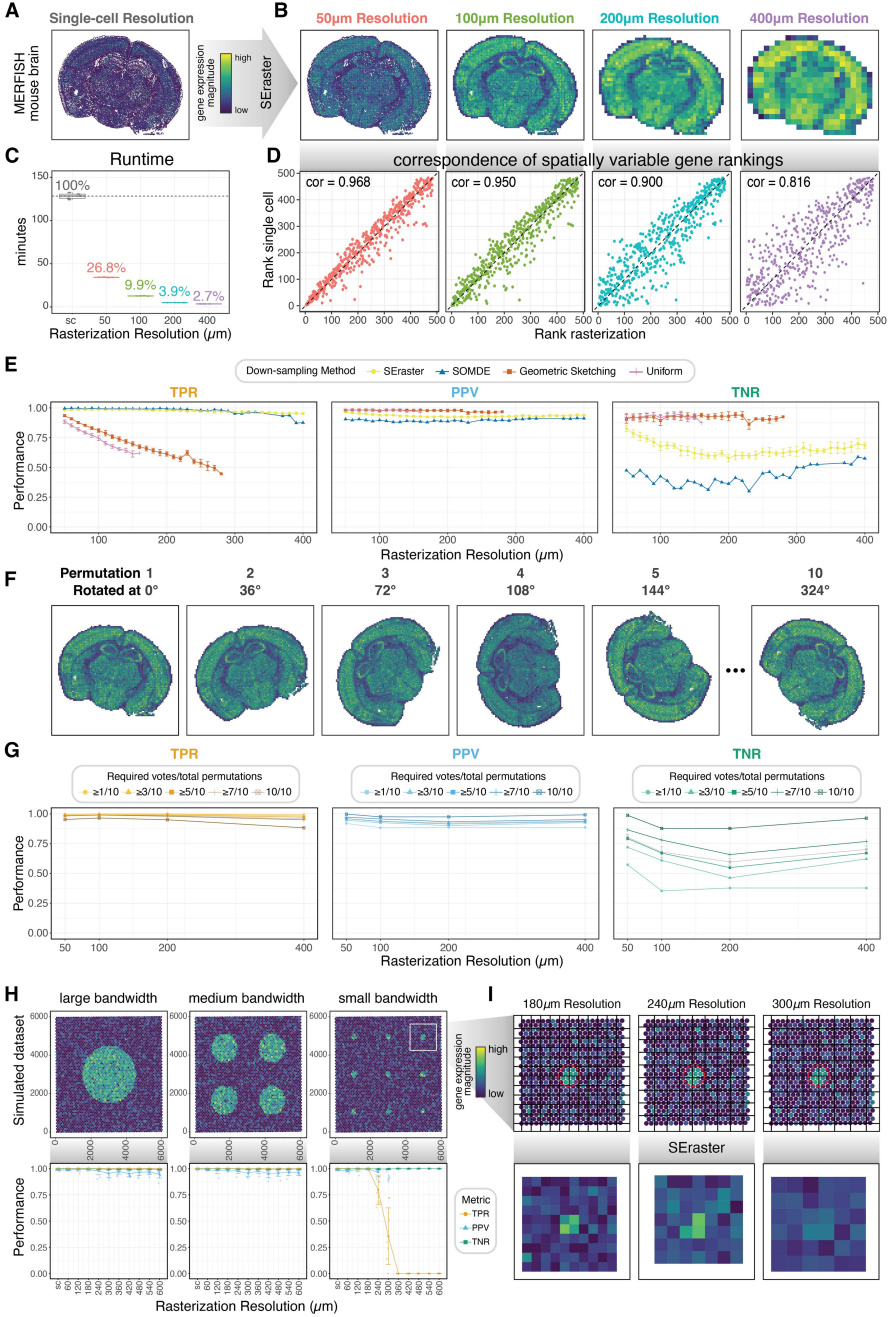
Impact of rasterization on the identification of spatially variable genes (SVGs). (A) Spatial transcriptomics dataset of a coronal section of mouse brain assayed by MERFISH shown at single-cell resolution colored by log-normalized total gene expression per cell. (B) MERFISH mouse brain dataset rasterized at 50, 100, 200, and 400 µm resolutions showing total rasterized gene expression per pixel (aggregated using mean). (C) Time (in min) required to run nnSVG at single-cell (sc) resolution and SEraster preprocessing with nnSVG at selected rasterized resolutions (n = 5 for each resolution) without parallelization (1 CPU core). Boxplots represent medians, first, and third quartiles, and whiskers extend to values no further than 1.5 times the interquartile range from each quartile. Percentage values of runtime at rasterized resolutions compared to that at single-cell resolution are shown. (D) Correspondence of gene rankings based on the estimated LR statistic from nnSVG at single-cell resolution and selected rasterized resolutions. Corresponding Spearman’s correlation coefficients are shown as text labels. (E) Performance comparison of SVG detection in terms of True Positive Rate (TPR), Positive Predictive Value (PPV), and True Negative Rate (TNR) for down-sampled (using SEraster, SOMDE, geometric sketching, or uniform sampling) MERFISH mouse brain data. Line plots show the mean and SD across 10 permutations for SEraster, geometric sketching, and uniform sampling methods. Line plots show the result of 1 permutation for SOMDE. NaN values are omitted. (F) MERFISH mouse brain data rasterized at 100 µm resolution and permutated across 10 angles. Plots are colored by total gene expression per pixel (aggregated using mean). (G) Performance comparison in terms of TPR, PPV, and TNR for rasterized (at 100 µm resolution) MERFISH mouse brain data using the voting method with 10 permutations. Colored line plots show performance metrics for the corresponding minimum required number of votes. Gray line plots indicate corresponding mean and SD across 10 permutations from (e) for comparison. (H) Simulated SVG dataset shown at single-cell resolution with large, medium, and small bandwidths showing log-normalized gene expression of ground truth SVG (top). Performance comparison in terms of TPR, PPV, and TNR for rasterized simulated data (bottom). Line plots show mean and SD across 10 permutations. NaN values are omitted. (I) Close-up visualizations of SVG with small bandwidth at single-cell resolution showing log-normalized gene expression and grid used upon rasterization at selected resolutions (top). Close-up visualizations of SVG with small bandwidth at selected resolutions showing rasterized gene expression (aggregated using mean) (bottom)

To evaluate the potential utility and impact of rasterization on the performance of downstream analysis, we focused on identifying SVGs within tissues. A number of computational tools have been previously developed to identify SVGs (Svensson *et al.* 2018, Sun *et al.* 2020, Kats *et al.* 2021, Miller *et al.* 2021, Zhu *et al.* 2021, Weber *et al.* 2023). We applied SEraster and one of these methods, nnSVG, to a single-cell resolution spatial transcriptomics dataset of a coronal section of the mouse brain containing 83 546 cells assayed by MERFISH (Fig. 2A, Supplementary Information S3I). We first evaluated the runtime of SVG analysis without parallelization (1 CPU core) when applied to the dataset at single-cell resolution (sc) vs rasterized at 50, 100, 200, and 400 µm resolutions (Fig. 2B). Combining SEraster and nnSVG reduced the total runtime to 26.8%, 9.9%, 3.9%, and 2.7% of that when running nnSVG at single-cell resolution for 50, 100, 200, and 400 µm rasterization resolutions, respectively (Fig. 2C, Supplementary Information S4). This shorter runtime is expected due to the fewer numbers of spatial points considered, particularly at coarser resolutions with larger pixel sizes (Supplementary Fig. S1A). Further, the contribution to runtime from SEraster preprocessing itself is minimal compared to the runtime of nnSVG (Supplementary Fig. S1B and C). Generally, SEraster preprocessing reduces runtime, though the extent of runtime reduction depends on the scalability of the downstream analysis tool with respect to the number of spatial points.

To evaluate the impact of SEraster on nnSVG results, we compared the ranks of each gene based on nnSVG’s likelihood statistics between single-cell and each rasterized resolution (Fig. 2D). Since nnSVG’s gene rankings indicate the strength of the spatial gene expression patterns, the observed high correlations of gene rankings suggests that the spatial pattern strengths of genes are generally retained even with SEraster preprocessing. We do observe comparatively lower correlations at coarser rasterization resolutions, suggesting that the forementioned relationships may be less well retained at coarser resolutions. We further characterized the performance of nnSVG in identifying SVGs when applied to rasterized gene expression by comparing SVGs identified at single-cell resolution, which were treated as ground truth, to those detected at each rasterized resolution (Supplementary Information S4).

We used this evaluation strategy to compare SEraster with other down-sampling approaches that can also be used to reduce the number of spatial points considered, including SOMDE, geometric sketching, and uniform down-sampling (Supplementary Information S4). Briefly, SOMDE integrates spatial information and applies self-organizing map to aggregate single-cell information into nodes (Hao *et al.* 2021). Geometric sketching integrates transcriptional information to select a subset of the original dataset while retaining its transcriptomic heterogeneity (Hie *et al.* 2019). Lastly, uniform or naïve down-sampling randomly selects a subset of the original dataset without taking neither spatial nor transcriptomic information into account. For SEraster, we observed that true positive rate (TPR) and positive predictive value (PPV)—also known as sensitivity and precision, respectively—remained high across rasterization resolutions, ranging from 0.96 to 0.99 for TPR and from 0.92 to 0.96 for PPV (Fig. 2E). SOMDE showed similar results, which is expected as SOMDE also aggregates single-cell information based on spatial information. TPR and PPV for SOMDE ranged from 0.87 to 1.00 and from 0.88 to 0.91, respectively (Fig. 2E). On the other hand, TPR for both geometric sketching and uniform sampling drastically decreased as down-sampled datasets contained fewer spatial points. For certain geometric sketching and uniform sampling down-sampled datasets, nnSVG failed, resulting in NaN values (Fig. 2E). However, among genes that were predicted to be SVGs for down-sampled datasets, both geometric sketching and uniform sampling retained high PPV ranging from 0.96 to 0.98 and from 0.96 to 0.99, respectively (Fig. 2E). Comparatively, spatially aware down-sampling methods performed worse in terms of TNR, or specificity. TNR for both geometric sketching and uniform sampling remained high ranging from 0.86 to 0.94 and from 0.88 to 0.95, respectively, while TNR for both SEraster and SOMDE were comparatively lower ranging from 0.58 to 0.83 and from 0.30 to 0.59, respectively (Fig. 2E).

To further explore the cause of these comparatively poorer performance with respective to TNR, we visualized false positives or genes misidentified as SVGs and saw they were primarily driven by hotspot artifacts, or small clusters (Supplementary Fig. S2A). To quantify this, we calculated the percentage of cells with nonzero expression and observed that false positive genes were all expressed by small proportions of cells whereas true positive genes were expressed by varying proportions of cells (Supplementary Fig. S2B). We hypothesized that if false positives were driven by hotspot artifacts, they would not be consistently misidentified across permutations, as hot spot artifacts would only happen at certain orientations of the tissue with respect to rasterization grids. Therefore, we sought to use permutations to improve our TNR. Briefly, we permuted the original dataset by rotating it at 10 different angles (Fig. 2F, Supplementary Information S2). Then, for each permuted dataset, we rasterized at selected resolutions and identified SVGs with nnSVG (Supplementary Information S4). Finally, we combined SVG classifications from the 10 independent results and determined genes to be SVGs if they were detected as SVGs in a minimum number of permutations, which we call required votes (Supplementary Information S4). As the number of required votes increased, TPR decreased slightly (Fig. 2G). For example, with at least one required vote, TPR ranges from 0.99 to 1.00, while with 10 required votes, TPR ranges from 0.88 to 0.97. This is because not all true positives are consistently detected as SVGs in all permuted grid orientations, which become false negatives when the number of required votes increases to become more restrictive. On the other hand, as the number of required votes increased, both PPV and TNR increased due to fewer false positives (Fig. 2G). For instance, with one required vote, PPV ranges from 0.88 to 0.92, and TNR ranges from 0.35 to 0.57. On the other hand, with 10 required votes, PPV ranges from 0.97 to 1.00, and TNR ranges from 0.88 to 0.99. This is because not all false positives are consistently detected as SVGs in all permuted grid orientations, which become true negatives when the number of required votes increases to become more restrictive. It is important to note that the magnitude of changes in TNR was much larger than those in TPR and PPV, which can be explained by asymmetric ground truth labels in this particular dataset with 401 SVGs and 82 non-SVGs predicted at single-cell resolution. Although the optimal number of required votes requires balancing various performance metrics and vary across datasets of interest, our results suggest that permutations can be an effective strategy for mitigating false positives to improve the TNR in SVG analysis with rasterization.

To further characterize the effects of rasterization resolution on nnSVG’s performance, we simulated spatial omics data with 100 SVGs and 900 noise genes across 4992 spatial points using a previously developed simulation framework (Supplementary Information S5i; Weber *et al.* 2023). By using simulations, we were able to modulate the scale of spatial patterns (large, medium, and small corresponding to circular spatial patterns for SVGs with radii of 1500, 750, and 150 µm, respectively) (Fig. 2H). We evaluated performance by comparing the simulated ground truth SVG or noise labels for each gene to those predicted by nnSVG when applied at single-cell vs at rasterized resolutions ranging from 60 to 600 µm (Supplementary Information S4). For simulated datasets with large and medium spatial patterns, nnSVG’s performance with rasterization remained high, with TPR values consistently at 1, PPV ranging from 0.95 to 1, and TNR ranging from 0.99 to 1 across evaluated resolutions (Fig. 2H). Notably, at all evaluated resolutions, the rasterized pixel size was smaller than the simulated SVGs’ circular spatial patterns with radii of 1500 and 750 µm. However, for simulated datasets with small spatial patterns, TPR started decreasing at 240 µm resolution and reached 0 at 360 µm resolution (Fig. 2H) due to nnSVG failing to detect any SVG at 300 µm and coarser resolutions, resulting in undefined PPV values and high TNR. These results are expected since the simulated SVGs’ circular spatial pattern has a radius of 150 µm. At coarser resolutions, the rasterized pixel size is too large such that cells with high expression of SVGs are aggregated with those with low expression of SVGs, eliminating signals (Fig. 2I). These findings suggest that users should choose a rasterization resolution that is sufficient to capture the size of spatial patterns of interest in order to maintain accuracy in their SVG analysis when using rasterization as a preprocessing step.

### 3.2 Rasterization enables scalable characterization of spatial cell-type co-enrichment

**Figure 3.**
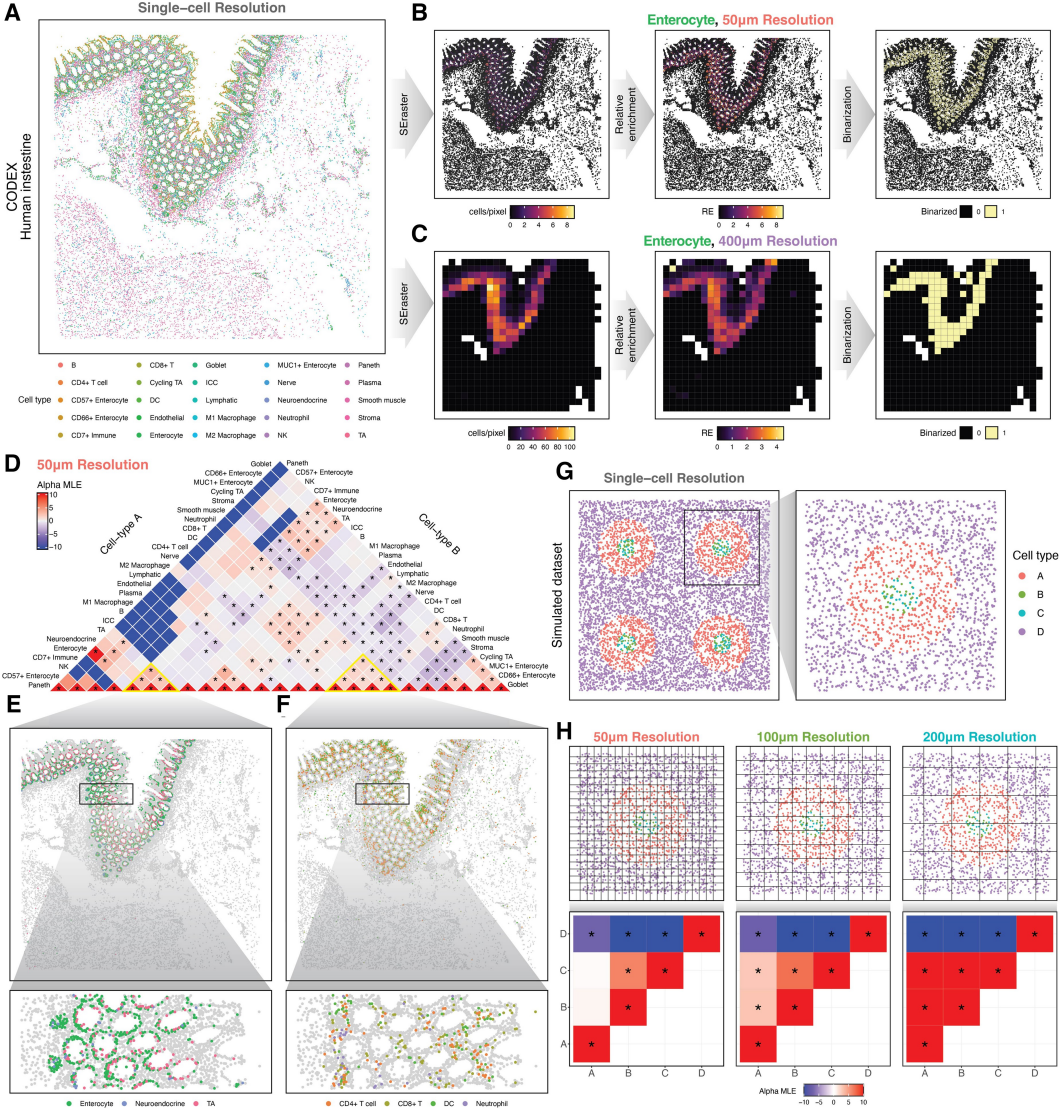
Rasterization of cell-type labels enables analysis of cell-type co-enrichment. (A) Spatial proteomics dataset of the human intestine assayed by CODEX shown at single-cell resolution with corresponding cell types. (B and C) Enterocytes in the CODEX human spleen dataset shown as rasterized cell-type count (aggregated using sum), relative enrichment (RE) metric, and binarized value per pixel at (B) 50 µm and (C) 400 µm resolutions (from left to right). (D) Summary of cell-type co-enrichment analysis with SEraster and CooccurrenceAffinity at 50 µm resolution. Heatmap shows the maximum likelihood estimate of the affinity metric (alpha MLE or $\hat{\alpha }$) for corresponding cell-type pairs. Statistically significant co-enrichments or depletions (adjusted *P*-value ≤.05) are indicated by asterisks (*). (E and F) Cell types with statistically significant co-enrichment ($\hat{\alpha }$>0, adjusted *P*-value ≤.05) at 50 µm visualized at single-cell resolution with corresponding cell types. (G) Simulated co-enrichment dataset shown at single-cell resolution with corresponding cell types. (H) Close-up visualizations of cell-type co-enrichment patterns at single-cell resolution with the grid used upon rasterization shown at selected resolutions (top). Summarized results of cell-type co-enrichment analysis with SEraster and CooccurrenceAffinity at selected rasterization resolutions. Heatmaps show $\hat{\alpha }$, and statistically significant co-enrichment or depletions (adjusted *P*-value ≤.05) are indicated by asterisks (*) (bottom)

To demonstrate additional potential applications of rasterization, we sought to use SEraster in identifying spatially co-enriched cell types. We applied SEraster to a single-cell resolution spatial proteomics dataset of a human intestine containing 38 371 cells assayed by CODEX (Fig. 3A, Supplementary Information S3ii). We used SEraster to aggregate cell counts for each cell-type within 50 µm pixels by rasterizing at 50 µm resolution. This allows us to treat rasterized cell-type counts in a classic balls (cell types) in boxes (50 µm pixels) framework to identify cell-type pairs that are co-enriched (Supplementary Information S6). Briefly, for each cell type, its rasterized cell-type count is used to compute a relative enrichment (RE) metric, or the ratio of observed to expected cell-type counts, per pixel to account for variability in cell density and cell-type proportions (Fig. 3B). Each pixel’s RE value is then binarized based on a selected threshold (Fig. 3B). Based on the binarized data, we apply the CooccurrenceAffinity package to compute the maximum likelihood estimate of the affinity metric, $\hat{\alpha }$, for each cell-type pair, with positive $\hat{\alpha }$ indicating co-enrichment and negative $\hat{\alpha }$ suggests depletion (Mainali and Slud 2022, Mainali *et al.* 2022). Using this approach, we identified cell-type pairs with statistically significant co-enrichment ($\hat{\alpha }$ > 0, adjusted *P*-value ≤.05**)** at 50 µm rasterization resolution that are consistent with previously identified tissue neighborhoods based on multi-scale neighborhood analysis (Hickey *et al.* 2023). For example, we identified enterocytes, neuroendocrine, and transit amplifying (TA) cells to be spatially co-enriched among each other (Fig. 3D and E), consistent with previously identified epithelial neighborhoods (Hickey *et al.* 2023). Likewise, we identified CD4+ T cells, dendritic cells (DC), CD8+ T cells, and neutrophils to be spatially co-enriched among each other (Fig. 3D and F), consistent with previously identified immune-related neighborhoods (Hickey *et al.* 2023). Notably, at 50 µm rasterization resolution, some immune- and epithelial-neighborhood-associated cell types are identified as significantly spatially depleted among each other ($\hat{\alpha }$ < 0, adjusted *P*-value ≤.05, Fig. 3D**)**.

To evaluate spatial co-enrichment relationships at a different length scale, we repeated this analysis rasterizing at 400 µm resolution (Fig. 3C). At 400 µm resolution, the immune- and epithelial-neighborhood-associated cell types previously identified as spatially depleted at 50 µm resolution are now identified as co-enriched (Supplementary Fig. S3). Notably, the immune- and epithelial-neighborhood-associated cell types still distinctly separate from endothelial, smooth muscle, and stromal cells, consistent with broad separations between the mucosa and submucosa/muscularis areas of the intestine (Supplementary Fig. S3B and C). These results suggest that choice of rasterization resolution can reveal spatial co-enrichment relationships at different length scales.

To further evaluate the impact of rasterization resolution on cell-type co-enrichment analysis, we employed a previously developed simulated dataset that mimics cell-type localizations at various scales (Peixoto *et al.* 2023). In this dataset, cell types B and C are spatially intermixed in circular spatial patterns with radius of 100 µm, and cell-type A surrounds cell types B and C in doughnut-shaped spatial patterns with radii ranging from 100 to 300 µm, separating them from cell-type D (Fig. 3G, Supplementary Information S5ii). At a rasterization resolution of 50 µm, cell types B and C had high, positive $\hat{\alpha }$ while A and B as well as A and C had $\hat{\alpha }\approx 0$ (Fig. 3G). This is expected because 50 µm resolution is smaller than the size of cell-type A’s doughnut-shaped structures. As a result, pixels did not capture the spatial pattern that cell-type A surrounds cell types B and C. With coarser resolutions—for instance, 100 and 200 µm resolutions that are within the range of cell-type A’s doughnut-shaped structures—pixel size is large enough to capture spatial patterns formed by cell types A, B, and C in one pixel. Thus, cell types A and B as well as A and C were identified as co-enriched (Fig. 3H). These results suggest that changing rasterization resolutions can capture cell-type co-enrichment relationships at various spatial length scales. Overall, SEraster can transform spatial omics data with cell-type labels into a classic balls-in-boxes formulation to enable characterization of cell-type co-enrichment.

### 3.3 Rasterization enables spatial analysis of spatial transcriptomics data of a whole mouse pup with over a million cells

**Figure 4.**
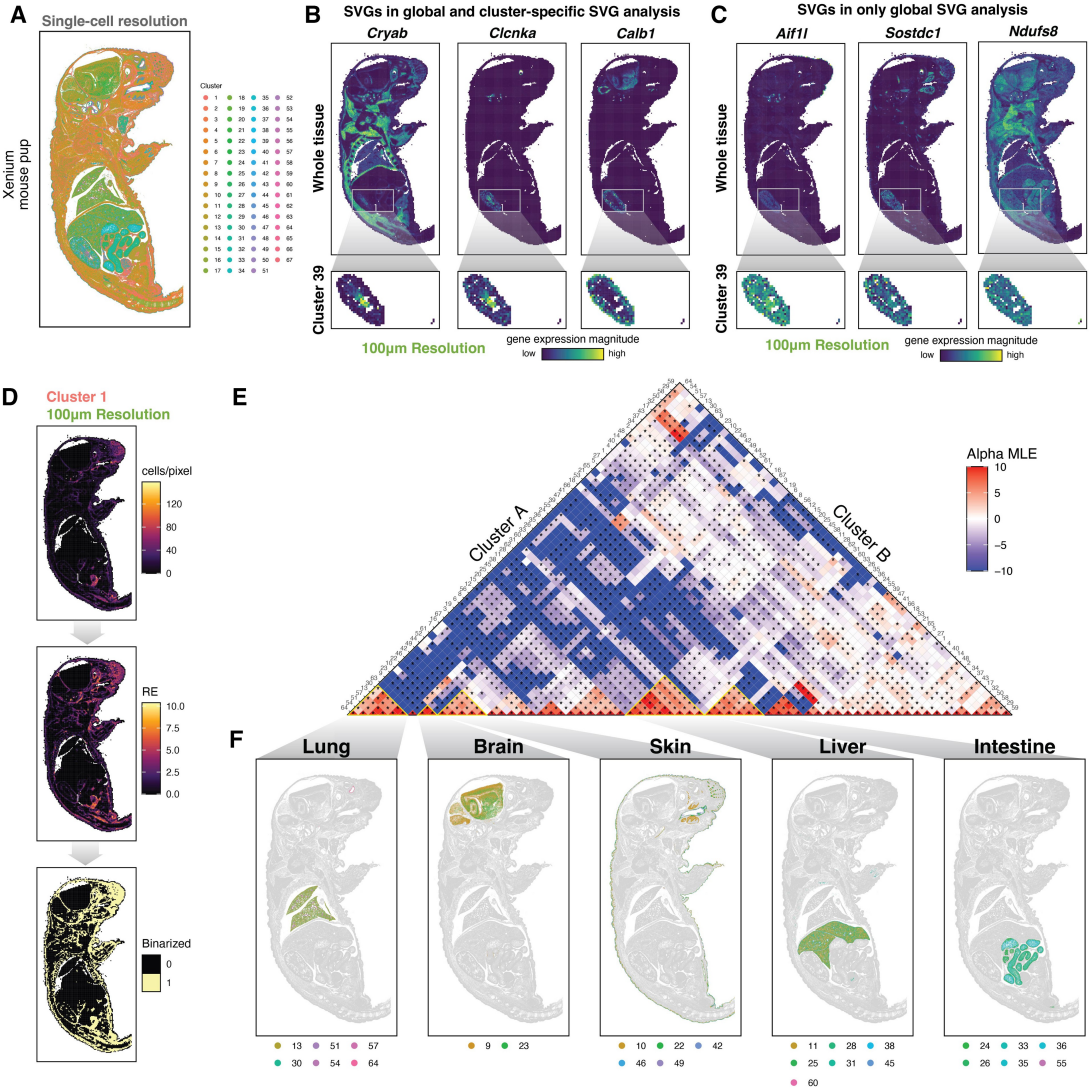
Spatial analysis of a whole mouse pup with over a million cells. (A) Spatial transcriptomics data of a whole mouse pup assayed by Xenium shown at single-cell resolution with corresponding clusters from graph-based clustering. (B) Rasterized gene expression (aggregated using mean) of *Cryab* (left), *Clcnka* (middle), *Calb1* (right) at 100 µm resolution for the whole tissue and within cluster 39. (C) Rasterized gene expression (aggregated using mean) of *Aifl1* (left), *Sostdc1* (middle), *Ndufs8* (right) at 100 µm resolution for the whole tissue and within cluster 39. (D) Cluster 1 in the Xenium whole mouse pup dataset shown as rasterized cluster count (aggregated using sum), relative enrichment (RE) metric, and binarized value per pixel at 100 µm resolution (from top to bottom). (E) Summary of cluster co-enrichment analysis with SEraster and CooccurrenceAffinity. Heatmap shows the maximum likelihood estimate of the affinity metric (alpha MLE or $\hat{\alpha }$) for corresponding cluster pairs. Statistically significant co-enrichments or depletions (adjusted *P*-value ≤.05) are indicated by asterisks (*). (F) Clusters with statistically significant co-enrichment ($\hat{\alpha }$>0, adjusted *P*-value ≤.05) at 50 µm visualized at single-cell resolution. Based on the spatial locations of co-enriched clusters, these co-enrichment patterns are labeled as lung, brain, skin, liver, and intestine (left to right)

Having demonstrated that our approach works as expected, we applied SEraster to a single-cell resolution spatial transcriptomics dataset of a whole mouse pup containing 1 330 087 cells assayed by Xenium (Fig. 4A, Supplementary Information S3iii). To identify SVGs, we again attempted to use nnSVG. However, running nnSVG, which scales linearly with the number of spatial points, at single-cell resolution failed to complete within 24 h (Supplementary Information S4). On the other hand, rasterizing the dataset to 100 µm resolution using SEraster and running nnSVG required an average total runtime of 54 ± 4 min without parallelization (1 CPU core) and 4 ± 0.1 min with parallelization (across 20 CPU cores) (n = 5, error is computed as SD, Supplementary Information S4), further highlighting the potential utility of SEraster in reducing computational resource requirements. We thus performed SVG analysis on the entire tissue rasterized at 100 µm resolution. Expectedly, all 379 profiled genes were identified as SVGs given that these genes were chosen to identify specific organs and tissue regions, which are highly spatially compartmentalized.

To better understand spatial gene expression variation within specific organs and tissue regions, we performed cluster-specific SVG analysis at 100 µm resolution for each transcriptionally distinct cell-cluster previously identified through graph-based transcriptional clustering (Supplementary Information S3iii and S4). As an example, we focused on Cluster 39, which putatively corresponds to the kidney based on its spatial location and differentially upregulated genes (Supplementary Information S3iii). Rasterizing just cells corresponding to cluster 39, we again performed SVG analysis to identify 118 SVGs. Among these SVGs included *Cryab*, *Clcnka*, *Calb1*, which exhibited statistically significant spatial variation (adjusted *P*-value ≤.05) both in the whole tissue analysis and cluster-specific analysis (Fig. 4B). On the other hand, genes such as *Aif1l*, *Sostdc1*, *Ndufs*8 only exhibited statistically significant spatial variation (adjusted *P*-value ≤.05) in the whole tissue analysis and not in the cluster-specific analysis as these genes exhibit more uniform expression within the cluster (Fig. 4C). These results demonstrate that SEraster can be used to help identify SVGs in the whole tissue as well as in a cluster-specific manner.

We further applied SEraster with CooccurrenceAffinity to characterize cell-cluster co-enrichment in the whole mouse pup at 100 µm resolution (Fig. 4D, Supplementary Information S6). We evaluated all 2278 possible cell-cluster pairs to identify 475 with statistically significant co-enrichment ($\hat{\alpha }$ > 0, adjusted *P*-value ≤.05**)** in 10 ± 1 min without parallelization (1 CPU core) and 1 ± 0.02 min with parallelization (across 20 CPU cores) (n = 5, error is computed as SD, Supplementary Information S4), again underscoring the scalability of our rasterization-based framework (Fig. 4). We further performed hierarchical clustering of $\hat{\alpha }$ values to find groups of cell-clusters that are co-enriched and visually correspond to spatially distinct organ structures (Fig. 3F, Supplementary Information S6). Further, we observed that such cell-cluster co-enrichment patterns forming spatially distinct organ structures are robust across rasterization resolutions ranging from 50 to 400 µm resolutions (Supplementary Fig. S4), demonstrating the stability of these particular cell-cluster co-enrichment relationships.

These results demonstrate that rasterization preprocessing with SEraster can be applied to large-scale spatial omics datasets with over a million single cells to enable the identification of SVGs at the whole tissue as well as cluster-specific levels and detection of cell-cluster co-enrichment patterns that correspond to spatially distinct organ structures.

## 4 Discussion

Analysis of spatial omics data provides researchers with means to delineate spatial patterns of molecular and cellular organizations. To improve the scalability of spatial omics data analysis, we developed SEraster to use rasterization as a preprocessing step to reduce the number of spatial points prior to downstream analysis by aggregating continuous variables, such as gene expression, or categorical variables, such as cell-type labels, at single-cell resolution into equally sized pixels based on a user-defined resolution. Such reduction in the number of spatial points enabled the spatial analysis of a whole mouse pup spatial omics dataset with over a million single cells using existing tools that would not have otherwise been computationally tractable. Applying SEraster prior to SVG analysis with nnSVG, we find that SEraster reduces runtime requirements without substantially compromising performance compared to single-cell resolution. Likewise, such SEraster preprocessing can achieve improved performance with respect to SVG analysis compared to other down-sampling approach, especially after integrating results from permutations. Finally, SEraster can also enable rapid, pair-wise cell-type co-enrichment analysis with CooccurrenceAffinity at multiple rasterization resolutions to explore cell-type spatial relationships across length scales.

While we exclusively examined single-cell resolution imaging-based spatial omics datasets in this paper, the same framework can, in principle, be applied to other non-single-cell resolution spatial omics data (Moffitt *et al.* 2022, Bressan *et al.* 2023), though interpretation of results will need to consider the lack of single-cell resolution and potential confounding due to cell-type mixtures. Likewise, although we have focused on applying SEraster to spatial transcriptomics and proteomics data, the framework can be applied to other spatial omics technologies as well.

Further, while we have focused on applying rasterization on individual spatial omics datasets, SEraster can also be applied to multiple tissue sections in order to create shared pixels in the same coordinate framework (Supplementary Information S1). For instance, such analysis can facilitate spatial molecular comparisons after structural alignment (Supplementary Fig. S5; Clifton *et al.* 2023). Such analyses can also be applied to assess molecular differences at structurally matched spatial locations across healthy and diseased tissues, though additional work is needed to characterize the statistical significance and biological relevance of identified differences while considering potential morphometric variation and errors in alignment for example.

Several limitations of SEraster should be considered when integrating SEraster with downstream analysis tools. As shown with SVG analysis on simulated datasets, rasterization resolution is a user-inputted hyperparameter that may affect downstream analysis. A rasterization resolution that is too coarse to capture the size of spatial patterns of interest will result in false negatives. Here, we draw a parallel between rasterization resolution and the concept of “grain size” in ecology (Wiens 1989), the spatial scale at which ecological processes are studied. As the choice of grain size will depend on the ecological phenomena of interest, so will the choice of rasterization resolution depend on the biological processes of interest. We recommend optimizing rasterization resolution based on prior knowledge regarding the scale of biological processes being studied. Potential future direction may involve leveraging rasterization resolution as an exploratory analysis tool. For example, SEraster can be performed in a series of various rasterized resolutions and characterize how the strengths of spatial expression patterns or length scales of SVGs change with respect to rasterization resolution.

In addition, SEraster offers a choice of mean or sum for aggregating single-cell information into pixels. By default, the mean function is used for rasterizing gene expression so that variability in cell density would not be a cofounding variable when performing downstream analyses, such as SVG analysis. For example, if sum is used as an aggregation function, regions with higher cell density would have higher rasterized gene expression even when a gene of interest is uniformly expressed by all cells in the tissue. Likewise, if sum is used, pixels that partially overlap with the tissue would have lower gene expression compared to those that fully overlap with the tissue even when a gene of interest is uniformly expressed by all cells in the tissue. These examples would yield false positives in the SVG analysis. For rasterizing cell-type labels, the sum function is used by default because the relative enrichment metric used in our co-enrichment analysis accounts for variability in cell density. We anticipate future work may involve expanding the range of aggregation functions to further include max, median, or weighted combinations as used in SOMDE (Hao *et al.* 2021) for tailored use cases. Additional characterization is necessary to determine whether these alternatives offer any advantages over mean or sum.

Although we have focused on demonstrating the utility of rasterization preprocessing with SEraster in downstream spatial omics analyses such as identifying SVGs and pair-wise cell-type co-enrichment, rasterization may also be amenable as a preprocessing step for other types of spatial omics analysis. For example, a few computational tools for inferring cell–cell communication have been developed for spatial transcriptomics data (Shao *et al.* 2022, Cang *et al.* 2023, Kim *et al.* 2023, Li *et al.* 2023). Potential future work may involve integrating these methods that leverage distributions of molecular information with SEraster to enable more scalable cell–cell communication analysis. However, we caution that additional statistical characterization is needed to benchmark performance and mitigate false positives.

In addition to the SpatialExperiment and Seurat objects that SEraster is built upon, there is a number of R data infrastructures with corresponding suites of exploratory analysis pipelines, such as the SpatialFeatureExperiment object for Voyager (Moses *et al.* 2023) and the Giotto object for Giotto Suite (Dries *et al.* 2021). Since SpatialFeatureExperiment is an extension of SpatialExperiment and a Giotto object can be converted into SpatialExperiment as well as vice versa, SEraster can potentially be integrated into a variety of analysis pipelines.

Finally, as spatial omics datasets continue to increase in size, in the future, we anticipate spatial omics datasets may need to be stored in standardized data infrastructure with lazy representation of larger-than-memory data such as the Zarr file format used in the SpatialData Python library (Marconato *et al.* 2024). Additionally, packages such as BPCells (Parks 2024), which uses bit-packing compression to store counts matrices as binary files on disk, are compatible with Seurat objects to perform memory-efficient analysis in R. SEraster can potentially be integrated with such data infrastructure to enable rasterization of larger-than-memory data in spatially indexed chunks rather than loading the entire dataset into memory.

To conclude, SEraster improves scalability by reducing the number of spatial points and broadens statistical methods for spatial omics data explorations. As spatial omics datasets continue to increase in the number of spatial points assayed, integrating such rasterization preprocessing with existing and new computational tools may enable more efficient analysis.

## Supplementary Material

btae412_Supplementary_Data

## Data Availability

The datasets underlying this article were derived from sources in the public domain and detailed in the Supplementary Information.
