# Supplementary material for: SEraster: a rasterization preprocessing framework for scalable spatial omics data analysis
Source: Bioinformatics. 2024 Jun 20;40(7):btae412. doi: 10.1093/bioinformatics/btae412 (PMC11226864; doi:10.1093/bioinformatics/btae412)
Supplement: btae412_Supplementary_Data [file btae412_supplementary_data.docx]

**Supplementary Information for SEraster: a rasterization preprocessing framework for scalable spatial omics data analysis**

**Supplementary Methods**

1. SEraster Overview

For both continuous variables, such as gene expression, and categorical variables, such as cell-type labels, the process of rasterization only differs by aggregation function. For a given spatial omics dataset with a features-by-observations matrix and x, y spatial coordinates, SEraster creates square or hexagonal grids. For square pixels, user-defined resolution corresponds to the side length of each pixel, and for hexagonal pixels, user-defined resolution corresponds to the distance between opposite edges of each pixel. The sf package (Pebesma et al., 2018) is used to create these square or hexagonal grids based on the bounding box including all spatial points in a given dataset as well as the surrounding space. Size of the surrounding space is approximately half of the user-defined resolution to ensure that all cells would be incorporated into square or hexagonal grids. Square pixels are used by default and all analyses in this particular work. SEraster’s rasterization is implemented in a pixel-wise manner, which is parallelized with the BiocParallel package. For each pixel with at least one cell, the features-by-observations matrix is subset to cells that reside within the pixel of interest, and their data is aggregated by mean or sum, essentially creating a features vector for the pixel of interest. At the end, a new features-by-observations matrix as well as x,y spatial coordinates of pixel centroids for rasterized dataset are returned as a SpatialExperiment object with the SpatialExperiment package.

Since both gene counts matrix and model matrix are sparse, they are often represented as sparse matrices to reduce memory requirements. Hence, SEraster can rasterize a SpatialExperiment object with the features-by-observations matrix represented as either dense or sparse matrix (dgCMatrix) using the Matrix package.

Further, SEraster can simultaneously rasterize multiple spatial omics datasets with shared pixel coordinates. To do so, SEraster creates square or hexagonal grids based on a common bounding box defined by minimum and maximum x,y spatial coordinates across given spatial omics datasets.

2. Permutation

To address the impact of specific locations and orientations of grids on downstream analysis, SEraster conducts permutation by rotating the single-cell resolution dataset at various angles, $\theta$, prior to rasterization with the following rotation matrix.

$$\left[ \begin{matrix} x' \\ y' \end{matrix} \right]=\left[ \begin{matrix} \cos\theta& -\sin\theta\\ \sin\theta& \cos\theta\end{matrix} \right]\left[ \begin{matrix} x \\ y \end{matrix} \right]$$

where $\theta$ is determined by a counterclockwise rotation from the x-axis around the midpoint of a two-dimensional Cartesian coordinate system.

3. Biological datasets

i. MERFISH mouse brain dataset

The MERFISH dataset of 1 mouse brain coronal section (slice 2, replicate 1) was obtained from the Vizgen website for *MERFISH Mouse Brain Receptor Map data release* (Vizgen, n.d.). “Blank” genes used for quality control were removed from the dataset. If cells did not have any gene with at least 2 RNA counts, they were considered as poor quality cells and were removed from the dataset. These filtering steps resulted in 83,546 cells and 483 genes. RNA counts were normalized by cell volume (divided per cell by the corresponding cell volume and scaled by the mean of all cell volume) as suggested when it is unknown whether the gene panel is skewed towards a particular cell-type or tissue region (Atta et al., 2024). Normalized counts were log_10_ transformed with a pseudocount of 1. Raw RNA counts, processed gene expression, and spatial coordinates were used to construct a SpatialExperiment object.

ii. CODEX human intestine dataset

The pre-processed CODEX datasets for human intestine tissue sections with labelled cell types, neighborhoods, communities, tissue units were obtained from Dryad (J. Hickey, 2023). As a proof of principle, we focused on the dataset corresponding to donor “B006” and tissue location “Ascending,” which contains 38,371 cells (J. Hickey, 2023; J. W. Hickey et al., 2023) for all analyses. Processed spatial coordinates, cell-type labels, neighborhood labels, community labels, and tissue unit labels were used to construct a SpatialExperiment object.

iii. 10X Genomics Xenium whole mouse pup dataset

The Xenium dataset of 1 day old mouse pup section was obtained from the 10X Genomics website for *Whole Mouse Pup Preview Data* (10X Genomics, 2023). Results for graph-based clustering analyses as well as differential gene expression analysis were also obtained from the same website. Genes used for quality control (those not included in the gene panel) were removed from the dataset. If cells did not have any gene with at least 2 RNA counts, they were considered as poor quality cells and were removed from the dataset. These filtering steps resulted in 1,330,087 cells and 379 genes. RNA counts were normalized by nucleus area (divided per cell by the corresponding nucleus area and scaled by the mean of all nucleus area) as suggested when it is unknown whether the gene panel is skewed towards a particular cell-type or tissue region (Atta et al., 2024). Normalized counts were log_10_ transformed with a pseudocount of 1. Raw RNA counts, processed gene expression, cluster labels (graph-based clustering), and spatial coordinates were used to construct a SpatialExperiment object.

Graph-based clustering labels were used for cluster-specific spatial variable gene (SVG) analysis, and an example of cluster-specific SVG analysis was shown using cluster 39. Cluster 39 was determined to likely correspond to kidney due to its location within the whole mouse pup tissue and differentially expressed genes (DEGs). Based on the DEG analysis performed by 10X Genomics via the Xenium Analysis Summary (10X Genomics, 2023), cluster 39 differentially upregulated *Lrp2*, a marker for proximal tubules (Khundmiri et al., 2021).

4. Spatial variable gene (SVG) analysis

SEraster aggregates gene expression of a given spatial omics dataset and creates a new SpatialExperiment object with a new features-by-observations matrix and x,y coordinates of pixel centroids. To conduct SVG analysis after rasterization with SEraster, this SpatialExperiment object is directly used as an input to the nnSVG package (Weber et al., 2022). For the whole tissue SVG analysis, SEraster rasterizes the entire dataset. For cell-type- or cluster-specific SVG analysis, the entire dataset is subsampled to a cell-type or cluster of interest, and SEraster rasterizes the subsampled dataset.

Runtime was measured using Sys.time function in the R Base package and averaged across 5 trials. While single-cell resolution quantified runtime for nnSVG alone, rasterization resolution quantified runtime for SEraster alone, nnSVG alone, and combined. This runtime analysis was done on a Mac Studio Apple M2 Ultra with 192GB unified memory (24‑core CPU, 60‑core GPU, 32‑core Neural Engine) using 1 or 20 workers for single computer multicore parallel evaluation (BiocParallel::MulticoreParam(workers = 1) or BiocParallel::MulticoreParam(workers = 20)).

For the MERFISH mouse brain dataset (rotated at 0°), gene rankings based on the estimated LR statistics computed within nnSVG were compared between single-cell resolution and selected rasterization resolutions. Spearman’s correlation coefficient was computed for each comparison.

Performance metrics were computed by comparing Boolean labels for each gene, indicating whether it is a SVG or not, between ground truth and selected rasterized resolutions. Since the nnSVG package computes multiple-testing-p-value using the likelihood ratio (LR) test with 2 degrees of freedom within itself (Weber et al., 2022), genes are considered as SVGs or to have statistically significant spatial variation based on a multiple-testing-p-value cutoff of 0.05 or below.

For the MERFISH mouse brain dataset, Boolean labels for the single-cell resolution were used as ground truths and compared with those for selected rasterized resolutions.

For the simulated SVG dataset, known Boolean labels were compared with those for the single-cell and rasterization resolutions. The following performance metrics were computed.

$$True Positive Rate \left( TPR \right)=\frac{TP}{TP+FN}$$

$$True Negative Rate \left( TNR \right)=\frac{TN}{TN+FP}$$

$$Positive Predictive Value \left( PPV \right)=\frac{TP}{TP+FP}$$

where $TP$, $TN$, $FP$, $FN$ denote the number of true positives, true negatives, false positives, and false negatives, respectively. To accommodate the sensitivity of rasterization from the orientation of grids with respect to the spatial omics data, performance metrics were summarized across 10 permutations rotated at 0°, 36°, 72°, 108°, 144°, 180°, 216°, 252°, 288°, and 324°.

The same approach for the MERFISH mouse brain dataset was used to compare SEraster with SOMDE (Hao et al., 2021), geometric sketching (Hie et al., 2019), and uniform or random sampling. For SOMDE, the number of spatial points or nodes after down-sampling must be $N\times N$, and SOMDE functions allow users to control the resulting number of nodes by choosing $k$, an expected average number of original spatial points each self-organizing map node represents. Thus, for each rasterized resolution, $k$ was determined to by performing an integer division of number of single-cells divided by the number of spatial points at a given rasterized resolution. Due to the SOMDE implementation, permutation was not used. We used Seurat’s implementation of geometric sketching (Seurat::SketchData) as described (Satija, 2023). For uniform or random sampling, we randomly down-sampled the dataset to a selected number of cells. For both geometric sketching and uniform sampling approaches, the number of spatial points after performing geometric sketching matched the number of spatial points for corresponding rasterized resolutions. In addition, instead of using rotation-based permutations, performance metrics were summarized across 10 permutations using 10 different random seeds with set.seed.

For the voting method, we permuted the MERFISH mouse brain dataset by rotating it at 0°, 36°, 72°, 108°, 144°, 180°, 216°, 252°, 288°, and 324°. Then, for each permuted dataset, we rasterized at 100 µm and identified SVGs with nnSVG as described above. Finally, we combined SVG classifications from the 10 independent results and determined genes to be SVGs if they were detected as SVGs in a minimum number of permutations, which we call required votes. 1, 3, 5, 7, 10 required votes were chosen for evaluation. Performance metrics for each required vote condition and selected rasterized resolutions were computed as described above.

5. Simulated datasets

i. nnSVG

Simulated datasets for SVG analysis were obtained from nnSVG (v1.5.8) (Weber et al., 2022). All datasets contained 4992 spatial points. These datasets simulated 100 SVGs and 900 noise genes based on the Visium human DLPFC dataset with 3 varying scales of spatial patterns (Weber et al., 2022). For all datasets, we scaled the spatial coordinates to 6,000 µm and 6,000 µm, which resulted in the scale of circular spatial patterns to have radii of 1500 µm, 750 µm, and 150 µm.

ii. CRAWDAD

The simulated dataset for cell-type co-enrichment analysis was obtained from CRAWDAD (v1.0) (Peixoto et al., 2023). This simulated dataset contains 8,000 cells that are randomly distributed across 2,000 µm x 2,000 µm space.

6. Cell-type co-enrichment analysis

SEraster uses a two-step binarization approach to create binary presence/absence data for a given spatial omics dataset. Relative enrichment (RE) metric represents the ratio of observed to expected cell-type counts, and similar framework has been used in the context of single-molecule localization microscopy for assessing colocalization of two molecular species with Voronoï tessellation (Ejdrup et al., 2022). In SEraster, RE for each pixel, $i$, and category, $j$, i computed using the rasterized cell-type or cluster labels based on the following equation

$$Relative Enrichment_{i,j} \left( {RE}_{i,j} \right)=\frac{observed_{i,j}}{expected_{i,j}}=\frac{observed_{i,j}}{n_{i}p_{j}}$$

where $n_{i}$ denotes the total number of cells in pixel $i$ and $p_{j}$ denotes the proportion of category $j$. Relative enrichment metric is then binarized based on a threshold of 1.

$$Binary_{i,j} \left( bin_{i,j} \right)=\left\{ \begin{aligned} 1, {RE}_{i,j}\geq1 \\ 0, {RE}_{i,j}<1 \end{aligned} \right.$$

This approach accommodates the differences in the proportion of cell-types and the cell density variance across spatial coordinates.

Binarized dataset is used to create 2 x 2 contingency tables indicating the presence and absence of corresponding cell-types.

|  |  | Cell-type A | |  |
| --- | --- | --- | --- | --- |
|  |  | Presence | Absence |  |
| Cell-type B | Presence | $k$ | $m_{B}-k$ | $m_{B}$ |
|  | Absence | $m_{A}-k$ | $N-m_{A}-m_{B}+k$ | $N-m_{B}$ |
|  |  | $m_{A}$ | $N-m_{A}$ |  |

where $k$ denotes the number of pixels with both cell-type A and B, $m_{A}$ denotes the number of pixels with only cell-type A, $m_{B}$ denotes the number of pixels with only cell-type B, and $N$ denotes the number of all pixels. The CooccurrenceAffinity package derived that the probability of cooccurrence under non-zero affinity, $\alpha$, between cell-type A and B follows the extended hypergeometric distribution (Mainali et al., 2022; Mainali & Slud, 2022).

$$P\left( X=k \right)=\frac{\binom{m_{A}}{k}\binom{N-m_{A}}{m_{B}-k}e^{\alpha k}}{\sum_{j=0}^{m_{B}} \binom{m_{A}}{j}\binom{N-m_{A}}{m_{B}-j}e^{\alpha j}}$$

$$\alpha=\log\frac{p_{1}(1-p_{2})}{p_{2}(1-p_{1})}$$

where $p_{1}$ denotes the probability that cell-type B occupies a pixel if cell-type A is present and $p_{2}$ denotes the probability that cell-type B occupies a pixel if cell-type A is absent.

For each contingency table, CooccurrenceAffinity (v1.0) computes a maximum likelihood estimate of affinity between a given cell-type pair, $\hat{\alpha}$, and test against the null hypothesis of $\alpha$ = 0 (Mainali et al., 2022; Mainali & Slud, 2022). Positive $\hat{\alpha}$ indicates co-enrichment, and negative $\hat{\alpha}$ indicates depletion. CooccurrenceAffinity applies an analytical null model based on hypergeometric distribution, resulting from the forementioned extended hypergeometric distribution when $\alpha$ = 0, instead of permutations, allowing rapid characterization of cell-type colocalizations. P-value associated with $\hat{\alpha}$ was determined within CooccurrenceAffinity based on Blaker’s “Acceptability” function (Mainali et al., 2022), and multiple testing correction using the Bonferroni correction was applied. Statistically significant co-enrichment between cell-type pairs were selected based on an adjusted p-value cutoff of 0.05 or below. Confidence intervals associated with $\hat{\alpha}$ were also computed within CooccurrenceAffinity using Blaker’s method (Mainali & Slud, 2022).

Hierarchical clustering of computed $\hat{\alpha}$ values represented as a $n_{j}$ x $n_{j}$ symmetrical matrix, where $n_{j}$ is the number of categories $j$, was done using the Heatmap function in the ComplexHeatmap package, which calls the hclust function in the stats package. “complete” argument was used for the agglomeration method.

Runtime for SEraster alone, cell-type co-enrichment analysis alone, and combined were measured using Sys.time function in the R Base package and averaged across 5 trials. This runtime analysis was done on Mac Studio Apple M2 Ultra with 192GB unified memory (24‑core CPU, 60‑core GPU, 32‑core Neural Engine) using 1 or 20 workers for single computer multicore parallel evaluation (BiocParallel::MulticoreParam(workers = 1) or BiocParallel::MulticoreParam(workers = 20)).

7. Computational implementation

SEraster is implemented as an R package within the Bioconductor framework. It builds on SpatialExperiment package (Righelli et al., 2022) for processing spatial omics data within the package, Simple Feature package (Pebesma et al., 2018) for executing rasterization, Matrix package (Bates et al., n.d.) for handling sparce matrix representations, and BiocParallel package (Morgan et al., 2023) for parallelization.

SEraster is also implemented as functions within the SeuratWrappers package, which extends Seurat functionality to rasterize spatial objects within Seurat’s spatial analysis framework (SeuratWrappers contributors, 2024). This implementation assumes that the Seurat Object already stores spatial coordinates and allows the user to specify the assay, slot, and/or image associated with the feature expression matrix or categorical metadata of interest when performing rasterization with the rasterizeMatrix function from SEraster. Results are output as a new object with an updated pixel-by-feature matrix, spatial coordinates, and associated metadata, which can then be analyzed and/or visualized using Seurat functions.

8. Code availability

SEraster is available as an R package on GitHub at <https://github.com/JEFworks-Lab/SEraster> with additional tutorials at <https://JEF.works/SEraster>. SEraster is also available as a SeuratWrappers on GitHub at <https://github.com/satijalab/seurat-wrappers>. Code to reproduce preprocessing, analyses, and figures in this manuscript is accessible on GitHub at <https://github.com/GohtaAihara/SEraster-analyses>.

**Supplementary Figures**

**
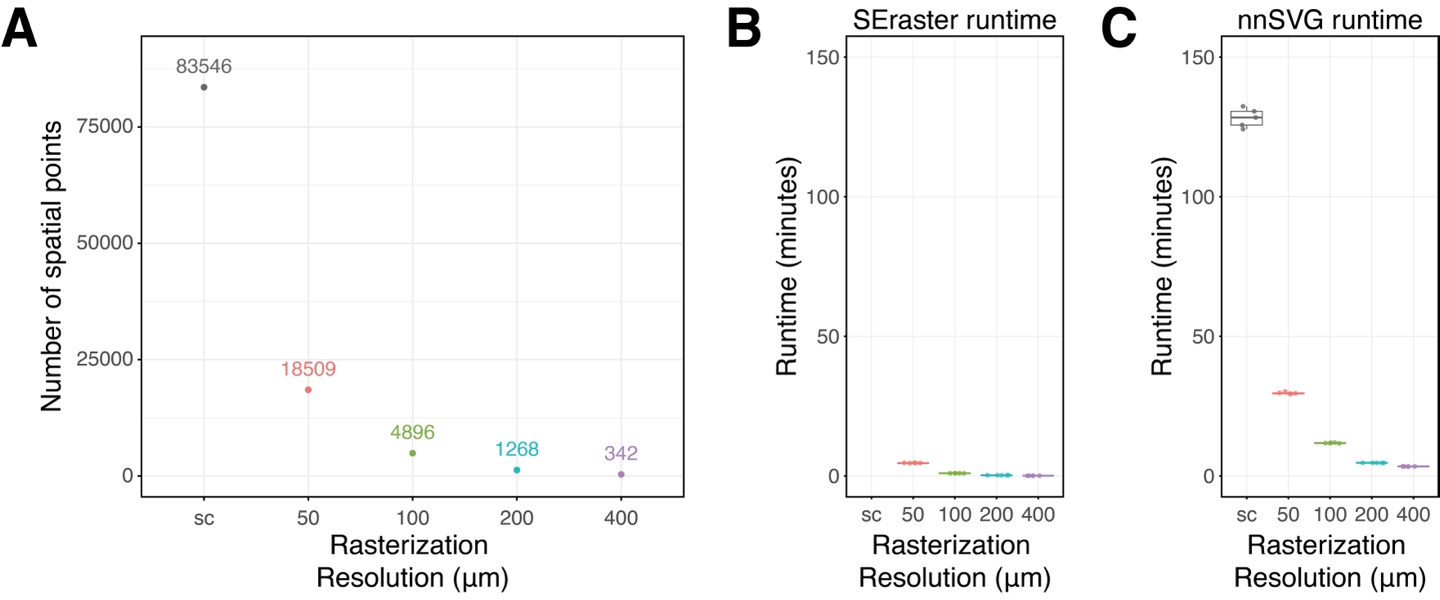
**

**Supplementary Figure 1. A.** Number of spatial points for the MERFISH mouse brain dataset at single-cell resolution (sc) and rasterized resolutions (50 µm, 100 µm, 200 µm, and 400 µm). Exact numbers of spatial points are shown as text labels. **B.** Time (in minutes) required to run SEraster preprocessing alone at selected rasterized resolutions ($n$ = 5 for each resolution). Boxplots represent medians, first, and third quartiles, and whiskers extend to values no further than 1.5 times the interquartile range from each quartile. **C.** Time (in minutes) required to run nnSVG alone at single-cell (sc) resolution and selected rasterized resolutions ($n$ = 5 for each resolution). Boxplots represent medians, first, and third quartiles, and whiskers extend to values no further than 1.5 times the interquartile range from each quartile.


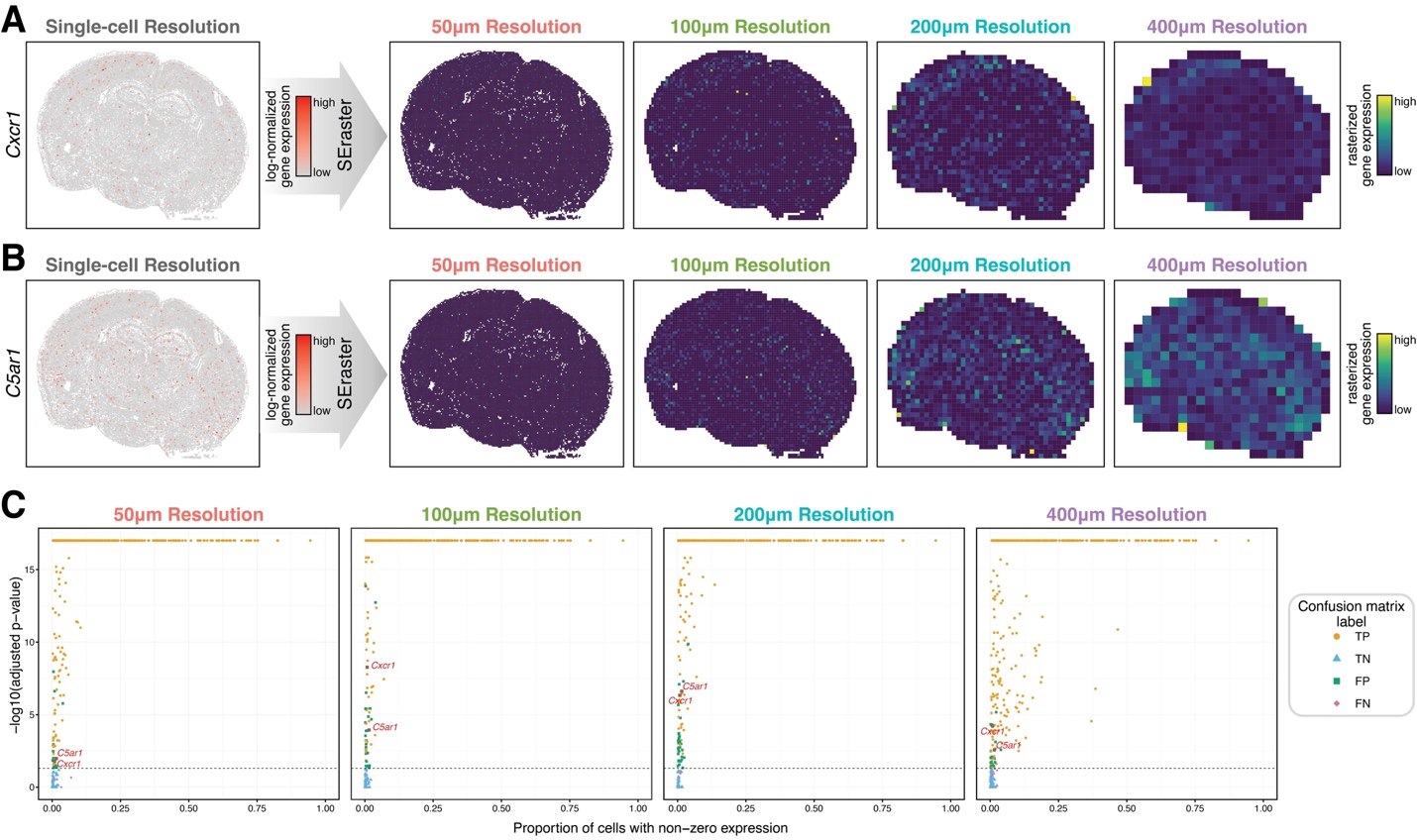


**Supplementary Figure 2. A-B.** Representative genes identified as false positive SVGs visualized at single-cell and rasterized (50 µm, 100 µm, 200 µm, and 400 µm) resolutions. Single-cell resolution plots are colored by log-normalized gene expression of (A) *Cxcr1* and (B) *C5ar1*, Rasterized resolution plots are colored by rasterized gene expression per pixel (aggregated using mean) of (A) *Cxcr1* and (B) *C5ar1*, **C.** The relationship between proportion of cells with non-zero expression and -log10(adjusted p-value) from nnSVG for each gene at 50 µm, 100 µm, 200 µm, and 400 µm resolutions. Genes are visualized with various colors and shapes based on their confusion matrix labels. *Cxcr1* and *C5ar1* are highlighted with red outlines. For genes with adjusted p-value = 0 or -log10(adjusted p-value) = Inf based on the nnSVG results, -log10(adjusted p-value) is set to 17 for better visualization.


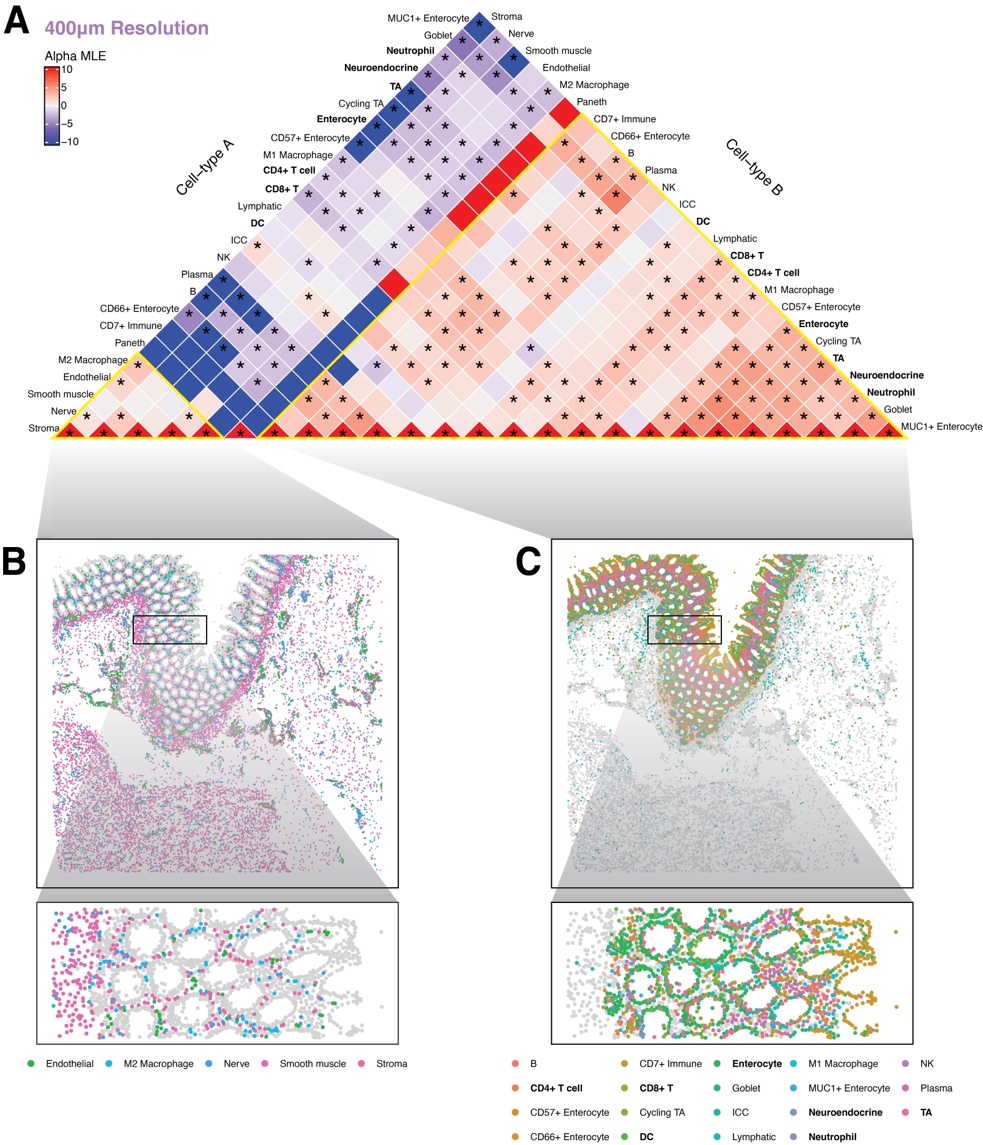


**Supplementary Figure 3. A.** Summary of cell-type co-enrichment analysis with SEraster and CooccurrenceAffinity at 400 µm resolution. Heatmap is colored by the maximum likelihood estimate of the affinity metric (alpha MLE or $\hat{\alpha}$) for corresponding cell-type pairs. Statistically significant co-enrichments or depletions (adjusted p-value ≤ 0.05) are indicated by asterisks (*). **B-C.** Cell-types with statistically significant co-enrichment ($\hat{\alpha}$ > 0, adjusted p-value ≤ 0.05) at 400 µm resolution visualized at single-cell resolution colored by cell-types. Cell-types previously identified as depleted at 50 µm resolution are bolded.


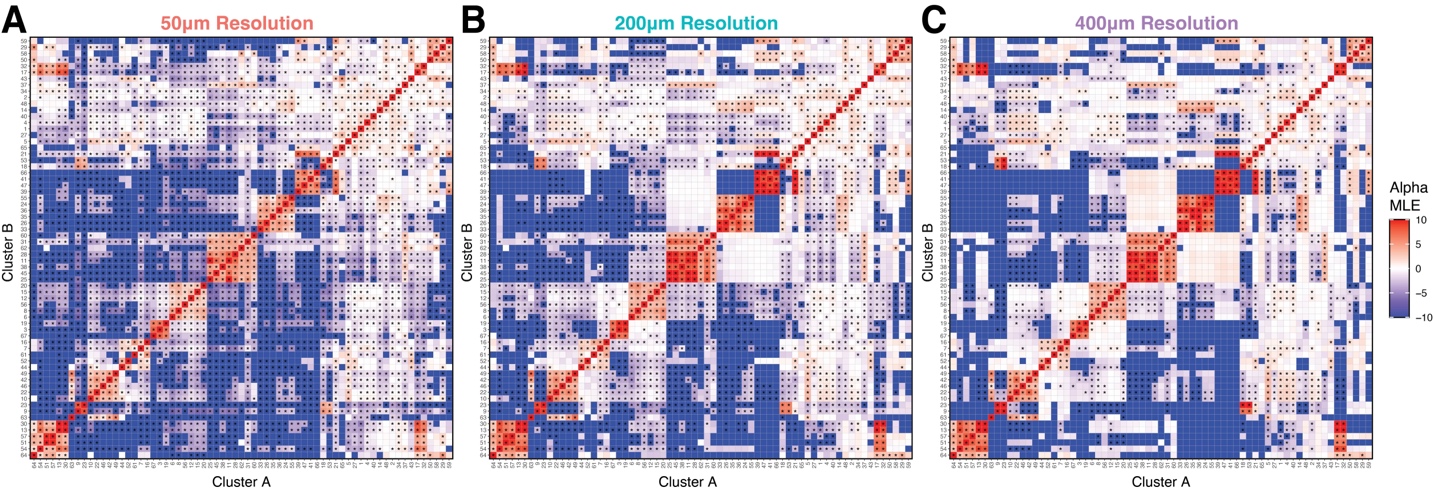


**Supplementary Figure 4.** Summary of cluster co-enrichment analysis for the whole mouse pup dataset with SEraster and CooccurrenceAffinity at **A.** 50 µm, **B.** 200 µm, and **C.** 400 µm resolutions. Heatmap is colored by the maximum likelihood estimate of the affinity metric (alpha MLE) for corresponding cluster pairs. Statistically significant co-enrichments or depletions (adjusted p-value ≤ 0.05) are indicated by asterisks (*).


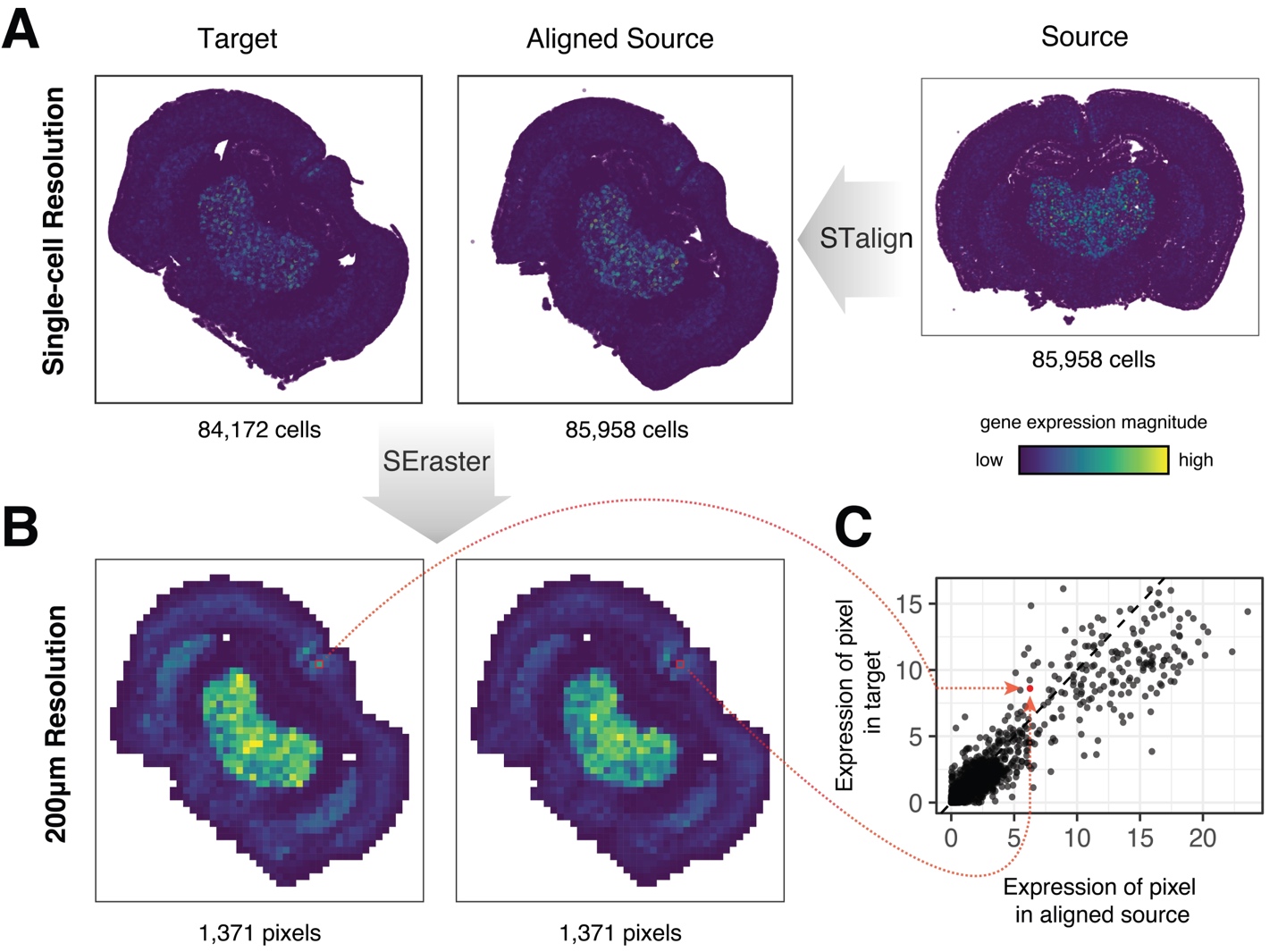


**Supplementary Figure 5. Application of SEraster to evaluate spatial correspondence of gene expression for validation of spatial alignment of single cell spatial transcriptomics datasets.** **A.** Alignment of source (right) and target (left) single-cell resolution spatial transcriptomics datasets of coronal sections of mouse brain assayed by MERFISH using STalign resulting in an aligned source (middle). Cells colored by expression of *Grm4.* **B**. Joint rasterization with SEraster of target (left) and aligned source (right) datasets at 200 µm resolution. Pixels colored by rasterized expression of *Grm4.* **C**. Correspondence of rasterized *Grm4* counts at matched pixel locations in target and aligned source. Example of matched pixel locations indicated in red.

**Supplementary References**

10X Genomics. (2023). *Whole Mouse Pup Preview Data (Xenium Mouse Tissue Atlassing Panel)*. https://www.10xgenomics.com/datasets/mouse-pup-preview-data-xenium-mouse-tissue-atlassing-panel-1-standard

Atta, L., Clifton, K., Anant, M., Aihara, G., & Fan, J. (2024). Gene count normalization in single-cell imaging-based spatially resolved transcriptomics. *Genome Biol*25, 153. https://doi.org/10.1186/s13059-024-03303-w

Bates, D., Maechler, M., Jagan, M., & Davis, T. (2024). *Matrix: Sparse and Dense Matrix Classes and Methods*. https://doi.org/10.32614/CRAN.package.Matrix

Ejdrup, A. L., Lycas, M. D., Lorenzen, N., Konomi, A., Herborg, F., Madsen, K. L., & Gether, U. (2022). A density-based enrichment measure for assessing colocalization in single-molecule localization microscopy data. *Nature Communications 2022 13:1*, *13*(1), 1–10. https://doi.org/10.1038/s41467-022-32064-y

Hao, M., Hua, K., & Zhang, X. (2021). SOMDE: a scalable method for identifying spatially variable genes with self-organizing map. *Bioinformatics*, *37*(23), 4392–4398. https://doi.org/10.1093/BIOINFORMATICS/BTAB471

Hickey, J. (2023). *Processed single cell data from CODEX multiplexed imaging of the human intestine*. https://datadryad.org/stash/dataset/doi:10.5061/dryad.pk0p2ngrf

Hickey, J. W., Becker, W. R., Nevins, S. A., Horning, A., Perez, A. E., Zhu, C., Zhu, B., Wei, B., Chiu, R., Chen, D. C., Cotter, D. L., Esplin, E. D., Weimer, A. K., Caraccio, C., Venkataraaman, V., Schürch, C. M., Black, S., Brbić, M., Cao, K., … Snyder, M. (2023). Organization of the human intestine at single-cell resolution. *Nature 2023 619:7970*, *619*(7970), 572–584. https://doi.org/10.1038/s41586-023-05915-x

Hie, B., Cho, H., DeMeo, B., Bryson, B., & Berger, B. (2019). Geometric Sketching Compactly Summarizes the Single-Cell Transcriptomic Landscape. *Cell Systems*, *8*(6), 483-493.e7. https://doi.org/10.1016/J.CELS.2019.05.003

Khundmiri, S. J., Chen, L., Lederer, E. D., Yang, C. R., & Knepper, M. A. (2021). Transcriptomes of major proximal tubule cell culture models. *Journal of the American Society of Nephrology*, *32*(1), 86–97. https://doi.org/10.1681/ASN.2020010009

Mainali, K. P., & Slud, E. (2022). CooccurrenceAffinity: An R package for computing a novel metric of affinity in co-occurrence data that corrects for pervasive errors in traditional indices. *BioRxiv*, 2022.11.01.514801. https://doi.org/10.1101/2022.11.01.514801

Mainali, K. P., Slud, E., Singer, M. C., & Fagan, W. F. (2022). A better index for analysis of co-occurrence and similarity. *Science Advances*, *8*(4), 9204. https://doi.org/10.1126/SCIADV.ABJ9204

Morgan, M., Wang, J., Obenchain, V., Lang, M., Thompson, R., Turaga, N., Lun, A., Bengtsson, H., Carlson, M., Atieno, P., & Oller, S. (2023). *BiocParallel: Bioconductor facilities for parallel evaluation*. https://bioconductor.org/packages/BiocParallel

Pebesma, E., Bivand, R., Racine, E., Sumner, M., Cook, I., Keitt, T., Lovelace, R., Wickham, H., Ooms, J., Müller, K., Pederson, T., Baston, D., & Dunnington, D. (2018). Simple Features for R: Standardized Support for Spatial Vector Data. *The R Journal*, *10*(1), 439–446.

Peixoto, R. dos S., Miller, B. F., Brusko, M. A., Atta, L., Anant, M., Atkinson, M. A., Brusko, T. M., Wasserfall, C. H., & Fan, J. (2023). Characterizing cell-type spatial relationships across length scales in spatially resolved omics data. *BioRxiv*, 2023.10.05.560733. https://doi.org/10.1101/2023.10.05.560733

Righelli, D., Weber, L. M., Crowell, H. L., Pardo, B., Collado-Torres, L., Ghazanfar, S., Lun, A. T. L., Hicks, S. C., & Risso, D. (2022). SpatialExperiment: infrastructure for spatially-resolved transcriptomics data in R using Bioconductor. *Bioinformatics*, *38*(11), 3128–3131. https://doi.org/10.1093/BIOINFORMATICS/BTAC299

Satija, R. (2023). *Sketch-based analysis in Seurat v5*. https://satijalab.org/seurat/articles/seurat5_sketch_analysis

SeuratWrappers contributors. (2024). *SeuratWrappers: Community-provided methods and extensions for Seurat*. https://github.com/satijalab/seurat-wrappers

Vizgen. (n.d.). *MERFISH Mouse Brain Receptor Map*. https://info.vizgen.com/mouse-brain-map

Weber, L. M., Saha, A., Datta, A., Hansen, K. D., & Hicks, S. C. (2022). nnSVG: scalable identification of spatially variable genes using nearest-neighbor Gaussian processes. *BioRxiv*, 2022.05.16.492124. https://doi.org/10.1101/2022.05.16.492124
